# A Case of Diseased Kidnies

**Published:** 1803-05-01

**Authors:** 

**Affiliations:** late of New Orleans


### *A* Case *of* diseased Kidnies, *communicated by Dr.* Dow, *late of New Orleans.*

A Person of a very sanguine temperament had been subject from his youth to frequent fits of giddines, occasioned by the afflux of blood to the brain. These attacks returning oftener, and with increased severity, at last became true suffusions of blood, accompanied with a sense of suffocation, redness in the face, and violent head-ach, so as to excite the apprehension of sanguineous apoplexy. In this situation he consulted a medical practitioner, who advised bleeding, which was performed immediately; and the patient found so much relief from it, that he afterwards constantly had recourse to the same operation whenever he felt the slightest head-ach or giddiness, without previously taking any medical advice to regulate the use of a remedy which is so extremely hazardous when carried to excess or ill employed.

This practice continued till he was about forty-two or three years old, without being followed by any serious ill consequence; only the fits of giddiness now became so frequent, that he was in the habit of being blooded sometimes twice or even thrice every month. At this time however he first began to remark that he was growing thin without any apparent cause, and he consulted his physician on the subject, who advised him to discontinue to lose blood, and to supply the place of this habitual evacuation by a liberal use of diluents, refrigerants, and a very guarded diet. The patient however neglected this advice, and still had recourse to blood-letting whenever he found it attended with temporary relief.

When he had reached the age of forty-five, several glandular enlargements appeared on the left side of the neck, and under the arm-pit on this side, at the same time that his emaciation increased, which symptoms began to alarm him so that he consulted several physicians separately, all of whom forbade the further use of bleeding, to to which they chiefly ascribed the disorders that had shewn themselves for some years past. The patient seemed to be determined to follow their advice, but at intervals he still continued his former fatal remedy, though he had been forewarned that it would infallibly bring him to his grave.

Some time after the above-mentioned period, the patient began to feel very severe pain in both hypochondria, particularly the left, and in the hypogastric region; at the same time he was attacked by a bilious fever, from which he was recovering with difficulty, when an empiric, who had gained his confidence, gave him so strong a dose of nitre, that it occasioned a violent fit of vomiting, together with intolerable pain in both hypochondria. Two hours afterwards a discharge of blood took place from the urethra, upon which the pain in the region of the kidnies ceased, bnt the bladder filled with coagulated blood, whilst the lower part of the abdomen became swelled and painful, and remained in this state till all the blood, extravasated into the bladder had come away in the form of long clots through the natural passage.

From this period the patient's health began gradually to decline, the mesenteric glands became obstructed, and, of course, the lacteals were unable to perform their office; the consequences of which were imperfect nutrition, and daily increasing emaciation. From this time also the hæmorrhagy from the kidnies returned with such frequency, that scarcely a month passed without two or more such accidents. He consulted several physicians, all of whom were agreed as to the diagnostic of the complaint, and the unfavourable prognostic which it afforded. It appeared to them evident that the frequent abuse of venæsection had insensibly deprived the blood of its balsamic part, that the lymph had thickened and stagnated in many of the glands, particularly those of the mesentery; that both the kidnies partook of the general obstruction, and that the pelvis of one, or perhaps both of them, contained some varicose veins, the frequent rupture of which was the cause of the hæmorrhagy above described. In consequence of this opinion, they only advised some general palliative remedies, giving up the hope of a radical cure.

The patient conformed to their prescription, but the frequent returns of the hæmorrhagy, together with some domestic uneasiness, so rapidly increased the disorder, and diminished the strength of the sufferer, that he was fast approaching to that state of emaciation and obstruction which which constitutes compleat marasmus; when both the hæmorrhagy and pain in the loins suddenly ceased.

This instant change gave the patient hopes of an immediate cure, and he began to form schemes of life, relying on an approaching state of good health; but this sudden amendment did not alter the opinion of the physician who then attended him; and the patient himself soon saw his illusive hopes disappear, when the pains returned with more violence than ever, in the glands of the neck and of the axilla, and in the arm on the same side, accompanied with such a sense of weight in the left thigh and leg, that they could hardly support the body in walking. Under these circumstances, he strongly importuned his physician to allow him to undergo a compleat course of mercury, believing that the cause of his complicated disorder might be some remains of a venereal taint in his constitution. This however the physician constantly opposed, as he could not perceive any indication of syphilis, and was apprehensive that the mercury, penetrating into several of the obstructed and schirrous glands, might dispose them to suppuration, and thus shorten the life of his patient.

However, the patient, strongly prepossessed with the opinion that nothing but mercury could save him, and importuned by his supposed friends, now put himself under the care of a young man, able in theory, but a bad practitioner; who, in the hopes of making a brilliant cure where his seniors had failed, entered into all the wishes of the sufferer, discovered several supposed symptoms of lues, took for venereal buboes all the indurated glands with which the patient's body was almost covered, and promised him a perfect cure; telling him, that if he had been called three months sooner, it would already have been compleated. He therefore, after due preparation, prescribed the compleat mercurial course, after the manner of M. Petit. The mercury, acting with violence on so dibilitated a subject, brought on such a severe salivation as to throw the patient into the last stage of marasmus; and even after this course had been discontinued, the pains returned with more frequency and acuteness; other glands became obstructed, and the patient could no longer forbear to complain to the young man of his mode of treatment. The latter replied that the spring, which was then approaching, was a more favourable season for cure than the winter, and proposed with confidence an immediate preparation for a second course of mercury, which, he said, could could not fail of success. The patient rejected this proposal with a degree of horror, dismissed the young man, and a week afterwards died suddenly.

The young man, to justify his practice, and, as he said, to prove the ignorance of those who had blamed his conduct, requested and obtained permission of the relations of the deceased to open the body. This was done in presence of the king's physician, and of the practitioner who had previously had the treatment of the case, who declared his opinion, before dissection, that the mesentery would be entirely obstructed, the kidnies in the same state, and perhaps suppurated.

The lungs were first examined; they were found rather turgid in their upper part, and contained some tubercles which had acquired a stony hardness. In the abdomen, the stomach and intestinal canal were found almost in a healthy state, only in a slight degree inflamed, the effect of the last moments of the deceased. The liver, the spleen, and the pancreas had undergone no alteration; the latter was raised much higher and more forward than its natural position, by a very bulky tumour situated beneath, the nature of which was not immediately understood, but, on careful examination, was found to be the left renal capsule, which had grown to a prodigious size and a very remarkable form. It covered, as usual, the upper part of the left kidney, rose above the middle of the spleen, then bent downwards towards the vertebral column, formed below the pancreas a kind of large bladder, twice as large as a turkey's egg, and at last became undistinguishably mixed with, and lost in, the mesenteric glands. It contained about a quart of a fluid, part of which was perfectly liquid, and the remainder nearly of the consistence and colour of wine lees.

The left kidney had enlarged to the size of a full grown cocoa nut, and was converted into a shapeless schirrous mass, so that it was only from its situation that it could be made out to be this viscus. Its pelvis was found entirely filled with an ill-conditioned pus, the sources of which were the infundibula of this cavity. No vestige of the emulgent vessels or of the ureter could be detected; every thing was confounded in one disorganized mass, in which the functions of this viscus had entirely ceased.

The right kidney had preserved its form, natural position, and texture; its bulk had increased about one-third, its pelvis contained urine, and the ureter, which was dilated and double its usual diameter, terminated in the bladder without any deviation. This enlargement of the kidney and ureter of the right side was owing to the disorganization of those on the opposite side, whose functions it had therefore to perform.

The right renal capsule had retained its natural situation, but had increased to the size of a turkey's egg. It contained a fluid similar to that of the left side.

The mesentery was in a state of general obstruction; all the glands were enlarged, from the size of a pea to that of a hen's egg, and the quantity of them was so great as to compress all the lacteals, and thus to prevent the absorption of nutritive fluid from the intestines.

The bladder was in every respect natural and healthy.

From this examination it appeared certain that no human assistance could have removed the disease, and that it was with reason that the patient had been advised to resort only to palliative remedies.

The above case likewise shews the danger attending a too frequent use of blood-letting, and the mischief to be apprehended from mercury, when symptoms similar to those above related have been brought on, either by the abuse of venæsection or by spontaneous hæmorrhagy.

